# Development and *In vivo* Pharmacokinetic and Pharmacodynamic Evaluation of an Oral Innovative Cyclodextrin Complexed Lipid Nanoparticles of Irbesartan Formulation for Enhanced Bioavailability

**DOI:** 10.7150/ntno.78102

**Published:** 2023-01-01

**Authors:** Narendar Dudhipala, Swetha Ettireddy, Ahmed Adel Ali Youssef, Goverdhan Puchchakayala

**Affiliations:** 1 Department of Pharmaceutics, Vaagdevi Pharmacy College, Warangal, Telangana, India - 506005.; 2 Synapse Life Sciences, Warangal, Telangana, India - 506001.; 3 Department of Pharmaceutical Technology, Faculty of Pharmacy, Kafrelsheikh University, Kafrelsheikh, 33516, Egypt.

**Keywords:** Irbesartan, Cyclodextrin, SLNs, Pharmacokinetic, Pharmacodynamic.

## Abstract

**Background:** Irbesartan (IR) is used in the treatment of hypertension, heart failure, and nephropathy in Type II diabetes. IR bioavailability is limited by poor solubility and presystemic metabolism. In our previous investigations, cyclodextrin (HPβCD) complexed solid lipid nanoparticles (SLNs) of IR were prepared, optimized, and characterized. The current study aimed to confirm the reproducibility of the previous methodology and to evaluate the pharmacokinetic (PK) and pharmacodynamic (PD) performance of the selected lead formulations in an experimental animal model.

**Methods:** SLNs were prepared by hot homogenization followed by probe sonication with IR/HPβCD inclusion complex loaded into a solid lipid (Dynasan 112). SLNs were evaluated for physical characteristics, drug content, entrapment efficiency, *in vitro* release profile, and surface morphology. The pharmacokinetic and pharmacodynamic behavior of the SLNs were evaluated in Wistar rats.

**Results:** Photon correlation spectroscopy, drug content, entrapment efficiency, and dissolution studies results were reproducible and consistent with our earlier investigation. PK studies showed 2.1-, 6.6-, and 9.9-fold improvement in the relative oral bioavailability of the drug from IR-HPβCD, IR-SLN, and IR-HPβCD-SLN formulations, respectively compared to IR suspension. However, IR-HPβCD-SLNs showed 1.5- and 4.7-fold improvement in the relative oral bioavailability of the drug compared to IR-SLN and IR-HPβCD formulations, respectively. PD studies in hypertensive Wistar rats showed a good control over systolic blood pressure for 48 h for SLN formulations compared to 2 h for IR suspension. However, the IR-HPβCD inclusion complex exhibited immediate antihypertensive activity (0.5 h) with a period of systolic blood pressure control similar to IR suspension.

**Conclusions:** The current approach exhibited improved oral bioavailability along with improved and prolonged pharmacodynamic effect.

## Introduction

Hypertension (HTN) is the most common cause of cardiovascular illness and premature global death 1. HTN affected 31.1% of the global adult population (1.38 billion people) in 2010 1,2. The prevalence of HTN among adults was higher in low- and middle-income countries (1.04 billion people, 31.5%) than in high-income countries (349 million people, 28.5%) 1,2. The global financial burden of HTN is estimated to be around 10% of overall global healthcare expenses 1,3. Therefore, HTN continues to be a worldwide public health challenge.

During the last four decades, worldwide mean blood pressure has remained constant or decreased slightly due to the widespread use of antihypertensive drugs 1. Irbesartan (IR) is an angiotensin II receptor antagonist, that inhibits the renin-angiotensin system by blocking the AT_1_ subtype of AII receptors 4. It is marketed under the trade name Aptovel^®^, Karvea^®^_,_ and Avapro^®^ by Sanofi-Aventis and Bristol-Myers Squibb 5. IR is used in the treatment of various diseases such as HTN, myocardial infarction, heart failure, and diabetic nephropathy 5. IR is classified based on the Biopharmaceutics Classification System (BCS) as a BCS class II drug (low aqueous solubility and high permeability) 6. The major drawbacks during the oral therapeutic application of IR are the low aqueous solubility (91.3 µg/ml) and the first-pass metabolism 7,8.

HPβCD came into existence in the pharmaceutical industry after the almost simultaneous patent by Janssen Pharmaceutica1 and the National Institutes of Health 9. It is a highly water-soluble βCD derivative, and it is an approved drug formulation excipient, included in both the USP-NF and EP 9. The glucopyranose units form a conical cylinder with a hydrophobic interior cavity and a hydrophilic outside surface. Inclusion complexation of drug molecules inside the interior cavity enhances the aqueous solubility of water-insoluble drugs, reduces photosensitivity, improves drug bioavailability (BA) across biological membranes, and provides good control on the release rates of lipophilic drugs as well 10,11.

Lipids have gained much attention as an emerging drug carrier approach to address many oral drug delivery challenges. In practice, lipid-based nanocarrier systems can be fabricated through blending ingredients such as pure triglyceride, mixed glycerides (e.g, Precirol^®^ ATO 5), lipophilic (e.g, Span^®^ 80) and/or hydrophilic surfactants (e.g, Tween^®^ 80), and water-soluble cosolvent 12. Encapsulating or solubilizing different drugs in the lipid excipients can improve solubilization and oral absorption, resulting in enhanced oral BA of the incorporated drugs 12. SLNs are submicron (50-1000 nm) spherical colloidal nanoparticles, with a drug-containing solid lipid core that remain in the solid state at room and body temperatures and are stabilized by surfactants 13,14. SLNs are used as an alternative drug carrier system for emulsions, liposomes, and polymeric nanoparticles 15. Drugs with poor oral BA due to low aqueous solubility in gastrointestinal tract fluids and/or first-pass metabolism can be loaded into SLNs, resulting in enhancing absorption into systemic circulation via the intestinal lymphatic transport and avoiding pre-systemic hepatic metabolism, thus enhancing the oral BA of drugs 14,16. Moreover, SLNs have several additional advantages, controlled release profiles and drug targeting, improved drug stability, high drug loading, the feasibility of loading lipophilic and hydrophilic actives, no biotoxicity of the carrier, and the feasibility of large-scale production and sterilization 15,17.

In our previous studies, we developed IR-HPβCD, IR-SLN, and IR-HPβCD-SLN formulations to enhance solubility, avoid pre-systemic hepatic metabolism, sustain release, and that could prolong the antihypertensive activity of IR through oral delivery. The dissolution efficiency was significantly increased with HPβCD complexation than with βCD complexation. Moreover, IR-SLNs and IR-HPβCD-SLNs showed a small particle size (<300 nm), better polydispersity index (<0.22), optimum values for zeta potential (-30.0 mV), high % entrapment efficiency (92.4±1.6), and showed sustained release of IR compared to the IR-CD (HPβCD and βCD) inclusion complexes, and IR suspension (IR-CS) over a period of 48 h 18. The current study aimed to extend our previous investigation to confirm formulation parameters' reproducibility and to evaluate the *in vivo* pharmacokinetic (PK) and pharmacodynamic (PD) performance of the lead formulations in normotensive and hypertension-induced rats, in comparison with IR-CS as control formulation.

## Materials & methods

### Materials

IR was a kind gift from Lupin Research Park Ltd., Pune, India. HPβCD (MW. 1500) was a generous gift from Dr. Reddy's Labs Pvt Ltd. (Hyderabad, Telangana, India). Tween^®^ 80, Dynasan 112, and Poloxamer 188 were procured from Sigma-Aldrich Chemicals (Bangalore, Karnataka, India). Soylecithin was a kind gift from Lipoid (Ludwigshafen am Rhein, Germany). Methanol and acetonitrile were of HPLC grade (Merck, Bengaluru, Karnataka, India). Centrisart filters (M.W. cut off 20,000 kD) were acquired from Sartorius (August-Spindler-Straße 11, 37079 Göttingen, Germany).

### Methods

#### Analysis of *in vitro* and *in vivo* samples

The concentration of IR was quantified based on a previously published reversed-phase HPLC-UV method with slight modifications and validated as in-house working conditions 19. The HPLC system consists of an LC- 20 AD solvent delivery unit with a reverse-phase Merck C18 (250 × 4.6 mm; 5 microns, Lichrosphere, Merck, Germany) column. The mobile consisting of phosphate buffer (10 mM, pH 3.0) and acetonitrile in a ratio of 55:45 was filtered through a 0.22 μm Millipore membrane filter, under vacuum, and degassed. The mobile phase was pumped isocratically at a flow rate of 1 mL/min. Peaks were obtained at a wavelength set at 244 nm using an SPD-20AV ultraviolet detector (SHIMADZU CORPORATION, Chiyoda-Ku, Tokyo, Japan). All samples were analyzed for IR at 25 °C, and a 50 μL injection volume was adjusted. The HPLC method was found to be linear over the IR concentration range of 0.08-50 µg/mL. The method was precise and accurate with a limit of detection (LOD) and limit of quantitation (LOQ) of 0.05 and 0.08 µg/mL, respectively.

#### Preparation of IR-HPβCD complex

The IR-HPβCD complex was prepared by the lyophilization method. A methanolic solution (30% v/v) of the drug was gradually added to the HPβCD solution in a ratio of 1: 4 (IR: HPβCD) under continuous stirring at 600 rpm for 6 h until a stable suspension was obtained. Then, drug suspension was converted to a clear solution by bath sonication. The solution was kept under continuous magnetic stirring to remove all solvent molecules. The obtained solutions were frozen at -20 °C and the frozen matrix was lyophilized in a freeze-dryer (Lyodel, Delvac Pumps Pvt. Ltd, Kanchipuram, Tamil Nadu, India) for 3 days to obtain a dry powder of IR-HPβCD complex.

#### Preparation of SLNs

Hot homogenization coupled with the ultrasonication method was employed in the preparation of IR-SLN and IR-HPβCD-SLN dispersion 20. The oily phase of IR-SLNs was prepared by dissolving IR (0.1% w/v), solid lipid (Dynasan 112, 2% w/v), and soylecithin (2% w/v) in chloroform and methanol mixture (5 ml, 1:1). A rotary evaporator (Heidolph, Schwabach, Germany) was then used to remove the organic solvents to prepare a dry layer containing IR. The dried layer was melted by heating above the lipid melting point by 5 ºC. It is worth mentioning that IR was added as an inclusion complex to the aqueous phase and was not added to the oily phase during the preparation of IR-HPβCD-SLNs. IR-HPβCD complex (52.6 mg, IR-HPβCD-SLNs) and/or Poloxamer 188 (1.5% w/v) were dissolved in double-distilled water to prepare the aqueous phase, which was heated to the same temperature as the oily phase. Then, the aqueous phase was added dropwise to the oily phase under continuous stirring for 5 min at 2,000 rpm to form a coarse emulsion. The coarse emulsion was then emulsified (Diax900, Heidolph, Germany, 16,000 rpm, 5 min) to further reduce particle size. The obtained O/W emulsion was then allowed to cool at 25 °C before subjecting to ultrasonication, using Vibra Cell Sonicator (SONICS^®^, CT, USA) equipped with a 12 T probe at 40% amplitude (10-sec pulse ON and 10-sec pulse OFF) for 25 min. SLNs were produced after allowing the obtained nanoemulsion to cool at room temperature.

#### Preparation of irbesartan suspension (IR-CS)

IR (10 mg) was triturated by the gradual addition of the suspending agent solution containing Tween^®^ 80 (1.0% w/v) to form a paste in a mortar. The paste was gradually diluted with double-distilled water to be easily transferred to a measuring cylinder and the final volume was adjusted. The dispersion was stirred (2000 rpm, 1 h at 40 ºC, then 1 h at 25 ºC) to form a uniform suspension.

#### Photon correlation spectroscopy (PCS)

The mean particle size (PS) and polydispersity index (PDI) of the size distribution of each formulation were determined by PCS using a Malvern Zetasizer (Nano ZS90, Malvern Panalytical Ltd, UK) at 25 °C in disposable folded capillary cells. The SLN dispersions were diluted at a 1:50 ratio with double-distilled water to yield a suitable scattering intensity. Analysis was conducted at 25 °C with an angle of detection of 90°. Zeta potential (ZP) was measured using the same diluted sample for each formulation 21,22. Each value was the average of three measurements.

#### Drug content

The IR content in the prepared SLN formulations was analyzed using the lipid precipitation method. The formulation (100 μL) was dissolved in a binary mixture of methanol and chloroform (900 μL, 1:1). Then, the mixture was vortexed for 5 min, centrifuged (Remi Laboratory Instruments, Mumbai, India) at 15,000 rpm for 20 mins, and the supernatant was collected for IR content analysis, following appropriate dilution with the mobile phase, using HPLC. The EE was calculated based on IR content.

#### Entrapment Efficiency (EE)

EE was determined by measuring the amount of the free (unentrapped) IR in the external phase based on earlier investigations 23. The continuous phase was separated by ultra-filtration technique using centrisart tubes. The centrisart tubes consisted of a filter membrane (MWCO, 20 KD), that divides the centrisart tube into two chambers (outer chamber and sample recovery chamber). A precisely measured volume of SLNs (2.5 ml) was placed in the outer chamber and centrifuged at 15000 rpm (15 min). During the filtration process, SLNs and the entrapped IR remained in the outer chamber, while the continuous phase containing the free IR moved to the sample recovery chamber. The amount of unentrapped IR was quantified using the HPLC method mentioned above. The % EE was calculated according to the following equation:



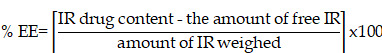



#### *In vitro* release testing for SLNs

The *in vitro* release testing was performed under sink conditions using vertical Franz cell apparatus (Logan Instruments, NJ, USA). Each vertical cell was consisting of a donor and receiver chamber. Dialysis membrane (MWCO; 12,000-14,000 kD, pore size; 2.4 nm, Hi-Media, Mumbai, India) was soaked in double-distilled water overnight before the release experiment. The dialysis membrane was clamped between the two half-cells of the diffusion cell. Phosphate buffer (pH=7.4) and 40% ethanol were used as the release medium 18. Nanodispersions (0.5 ml) were added to the donor compartment, and the receiver compartment had 12 ml of a freshly prepared dissolution medium. The study was carried out at 37±0.5 ºC. At predetermined time points (t=0, 0.5, 1, 2, 3, 4, 6, 8, 10, 12, 24, and 48 h), aliquots (1 mL) were withdrawn from the receiver chamber and an equal volume of the dissolution medium was replaced in the receiver compartment after every sample withdrawal. Samples were analyzed using the HPLC to quantify the amount of IR released across the dialysis membrane at each time point.

#### Scanning electron microscopy (SEM) analysis of IR-HPβCD

Morphology of the freeze-dried IR-HPβCD inclusion complex and IR-HPβCD physical mixture was compared against the physical appearance of the pure IR using a Hitachi S-520 scanning electron microscope (Hitachi, Tokyo, Japan). Using a double-face adhesive carbon tape, SEM samples were mounted onto an aluminum stage. Samples were then gold sputter-coated under an argon atmosphere in a high vacuum evaporator to achieve a uniform coating without any thermal damage and to render them electrically conductive. The images were captured using a scanning electron microscope, operating at an accelerating voltage of 3-10 kV 24.

#### Transmission electron microscopy (TEM) analysis of SLNs

TEM analysis was performed using a JEOL-100CX-II instrument (Tokyo, Japan). TEM samples (IR-SLNs and IR-HPβCD -SLNs) were examined according to a negative staining protocol with a solution of sodium phosphotungstate (0.2% w/v). A carbon-plated copper grid was placed above a 20 µL drop of the nanocarrier dispersion for 90 sec and after removal, the excess sample was removed with the aid of a filter paper. Then, the grid was placed above a 20 µL drop of the staining solution for 10 sec, and after removal, the excess stain was drawn off the grid with the aid of a filter paper. Next, the grid was allowed to dry for a few minutes by air. Later, the grids were examined under the transmission electron microscope at 25K times magnification power 25.

### Pharmacokinetic (PK) Studies

#### Study design and sampling protocol

Albino male Wistar rats (200 ± 30 g) were selected for the study. Rats were housed in well-ventilated polypropylene cages under standard laboratory conditions (25 °C, 55% RH) with free access to a standard diet and water *ad libitum*. All studies were performed according to Committee for the Purpose of Control and Supervision of Experiments on Animals (CPCSEA) guidelines (Department of Animal Welfare, Ballabhgarh, Haryana, India) after approval from the Institutional Animal Care and Use Committee (IACUC, Synapse life sciences, Warangal, India, 1891/PO/Re/S/17/CPCSEA; #SLS/02/10/2017). Under fasting conditions, IR-HPβCD, IR-CS, IR-SLN, and IR-HPβCD -SLN formulations (Dose; 10 mg/kg) were administered to Wistar rats, randomly assigned to 4 groups (n=6 for each group) in a single dose BA study. After dosing, blood samples (0.5 mL) were collected into Eppendorf tubes by retro-orbital plexus puncture at predetermined intervals (0, 0.5, 1, 2, 3, 4, 6, 8, 10, 12, and 24 h) under light anesthesia. Blood samples were set aside undisturbed for 20 minutes to clot at room temperature. Next, blood samples were centrifuged for 30 min at 3000 rpm at room temperature. Then, the supernatant (serum, 200 µL) was transferred to another Eppendorf tube and stored at -20 °C until analysis by the HPLC method described above.

#### Extraction of IR from rat serum

All stored samples (rat serum) were thawed before analysis. Briefly, 100 μL of telmisartan solution as an internal standard (5.0 μg/mL) were added to 100 µL of the sample in Eppendorf tubes and vortexed for 3 min. Then, acetonitrile (300 µL) was added as a protein precipitating agent and the mixture was vortexed again for 3 min. Next, methanol (300 µL) was added as an extracting solvent, and the mixture was vortexed for another 5 min. Later, the mixture was centrifuged at 10000 rpm for 10 min. The clear supernatant was removed from the HPLC vial and 50 μL was injected into the HPLC system for IR quantification 19.

#### Calculation of PK parameters

After a single oral dose, PK parameters of IR-HPβCD, IR-CS, IR-SLN, and IR-HPβCD -SLN formulations such as t_max_ (time for peak serum concentration), AUC_total_ (area under the plasma drug concentration-time curve from time zero to infinity), t_½_ (biological half-life), MRT (mean residence time), and C_max_ (peak serum concentration) were calculated via non-compartmental analysis using Kinetica software (version, 5.0).

### Pharmacodynamic (PD) studies in untreated and hypertensive rats

#### Study design and sampling protocol

Albino male Wistar rats (180 ± 30 g) were given free access to a standard laboratory diet and drinking tap water to acclimatize animals for 7 days to the environment of the experiment. During these 7 days, the rats were taught to remain calm and non-aggressive in the rat holder. The initial weight and the systolic blood pressure (SBP) of the individual rats were estimated before the induction of HTN by replacing the drinking tap water with fructose solution (10% w/v) for two weeks. After these two weeks, rats exhibiting a minimum mean SBP of 140-145 mmHg were screened as hypertensive rats and used for the PD study. The hypertensive rats were divided into five groups, I, II, III, and IV, treated orally with IR-CS, IR-HPβCD, IR-SLN, and IR-HPβCD-SLN formulations (Dose; 10 mg/kg), respectively, while group V served as control, and each group consisted of six rats 26. SBP was measured at predetermined time intervals (0, 0.5, 1, 2, 4, 8, 12, 24, 36, 48, 72, 96, and 120 h) and concurrently measured for untreated rats also (group VI).

#### Tail-cuff method

SBP was measured by applying the Tail-cuff method. Rats were housed in plastic holders before being placed in temperature-controlled chambers. A cuff with a pneumatic pulse sensor was attached to the rat tail. However, rats were given two days to get used to the experiment before SBP measurements. On an NIBP, IITC 59/29 model, SBP readings were recorded 27. At least three readings were obtained from each rat.

### Statistical analysis

Graph pad prism software (version 5.02.2013, San Diego, CA, USA) was employed for the statistical analysis of data, and a statistically significant difference was reported based on a p-value less than 0.05.

## Results and Discussion

### Composition of IR-SLNs and IR-HPβCD-SLNs

The composition of SLN formulations is shown in **Table 1**. The physicochemical stability experiment indicated that IR-SLN and IR-HPβCD-SLN formulations were stable for 2 months during room temperature storage. Many drug delivery literature proved that not only electrostatic repulsion improved the stability of lipid nanoformulations, but the use of steric stabilizers such as Poloxamer 188 and soylecithin can also increase the electrostatic stabilization by reducing electrostatic repulsion between the lipid nanoparticles by forming a coat around the SLN surface 28,29.

### Physicochemical characterization of SLNs

Presystemic hepatic metabolism presents as a formidable hurdle to drugs suffering from the first-pass effect. Formulation scientists in academia and industry are looking for alternative pathways for drug transport to thwart problems related to the portal vein-to-liver pathway. Intestinal lymphatic transport is an emerging promising pathway for oral drug delivery to struggle against this obstacle. Compared with the portal vein-to-liver pathway, lymphatic transport delivers the drug directly to the systemic circulation through the lymphatics and thus avoiding hepatic first-pass metabolism, allowing transport of relatively large-size macromolecules through the leaky capillaries of the lymphatic system, and holds potential for the treatment of diseases afflicting the lymphatic systems 30. The small PS of nanocarriers (20-500 nm) allows efficient uptake of drug molecules via the lymphatic route into the intestine 31.

Physicochemical characteristics of the prepared SLNs are depicted in **Table 2**. Nanocarriers with a PDI value of 0.3 or below is deemed acceptable and correspond to homogeneous and monodispersed system 32. One of the most popular uses of ZP data is to relate it with colloid stability. Although ZP provides insight on colloid stability, it does not reveal the entire picture. However, nanodispersion with ZP values of ˃ ± 30 mV is considered highly stable according to drug delivery literature 32. SLN formulations showed PS < 500 nm, moderately dispersed systems (PDI, 0.1-0.4), and high stability (-30 mV). The drug content of IR in IR-SLN and IR-HPβCD-SLN formulations was 98.3±2.1 and 99.8±2.5%, respectively. Moreover, the IR-SLN and IR-HP-βCD-SLN formulations showed high EE values of 90.2±1.9 and 92.4±1.6%, respectively. There was no significant difference (p>0.05) observed for the PS, PDI, ZP, drug content, and EE values compared with the earlier published investigation 18. Dynasan-112 has C12 triglyceride branched chains that could produce a lipid matrix with a lot of imperfections that can accommodate more drug molecules which is advantageous to increase IR loading capacity 27,33. This observation came in accordance with earlier reported studies 27.

### *In vitro* release studies for SLNs

**Figure 1:**
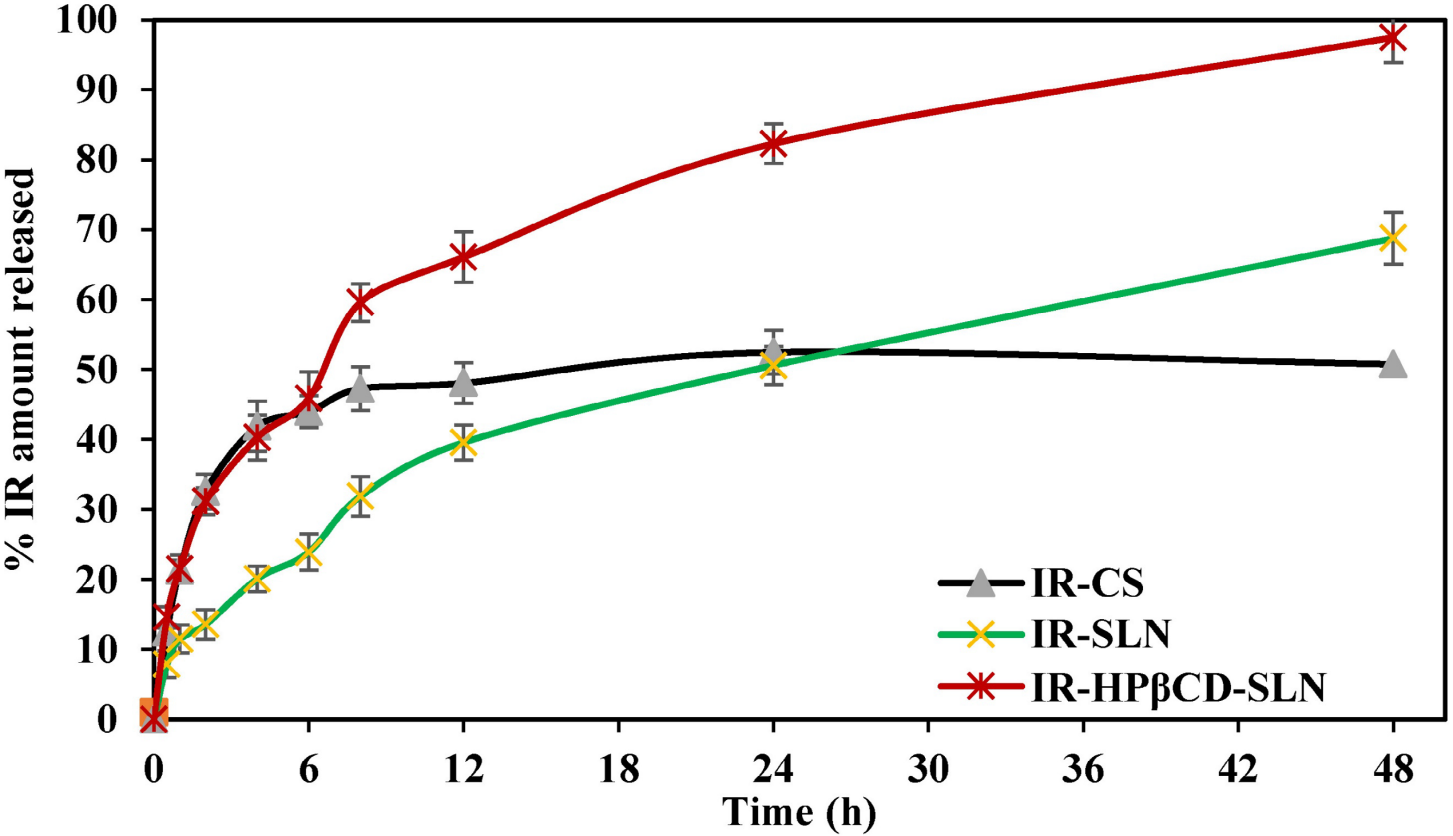
The *in vitro* release profiles of irbesartan from IR-CS, IR-SLN, and IR-HPβCD-SLN formulations (mean±SD, n=4).

Phosphate buffer (pH 7.4) with 40% ethanol was used as the release medium for *in vitro* release testing for 48 days by the dialysis method 34. According to the United States Pharmacopeia (USP), organic cosolvents such as ethanol are routinely added as solubility modifiers to the aqueous dissolution media to afford sink conditions. The release profiles are shown in **Fig. 1**. A sustained release profile of IR was observed from all formulations. IR-CS showed a quite fast dissolution rate during the first 4 h because the fraction of free IR in solution was immediately ready to diffuse through the release medium; however, the release profile rapidly reached a plateau phase during the next 44 h, giving rise to a maximum of 50.8±2.9% drug released, whereas SLNs exhibited relatively high drug release 67.8±3.7 and 97.5±3.6% for IR-SLN and IR-HPβCD-SLN, respectively. The results showed a slight but non-significant difference (p>0.05) compared to our previous evaluation 18. The sustained release characteristic of all formulations could be due to the fact that IR is a lipophilic drug belonging to the BCS class-II. In addition, IR-HPβCD-SLN exhibited the highest % drug release in comparison to the IR suspension and IR-SLN containing IR alone, this could be due to the wetting and solubilizing effect of HPβCD towards IR 35. Moreover, the increase in drug release rate was more evident in the case of SLNs in comparison to IR-CS, giving rise to a high % drug release. This finding could be due to the formation of a drug containing supercooled melts with an amorphous nature at room temperature. It was observed in earlier investigations that Dynasan-based nanocarriers could either exist in either the solid crystalline state or the supercooled liquid state at room temperature. Supercooled melt behaves like O/W emulsions (liquid state), suggesting that the diffusion of drug molecules through the supercooled melts is expected to occur easily 36.

### SEM analysis

**Figure 2:**
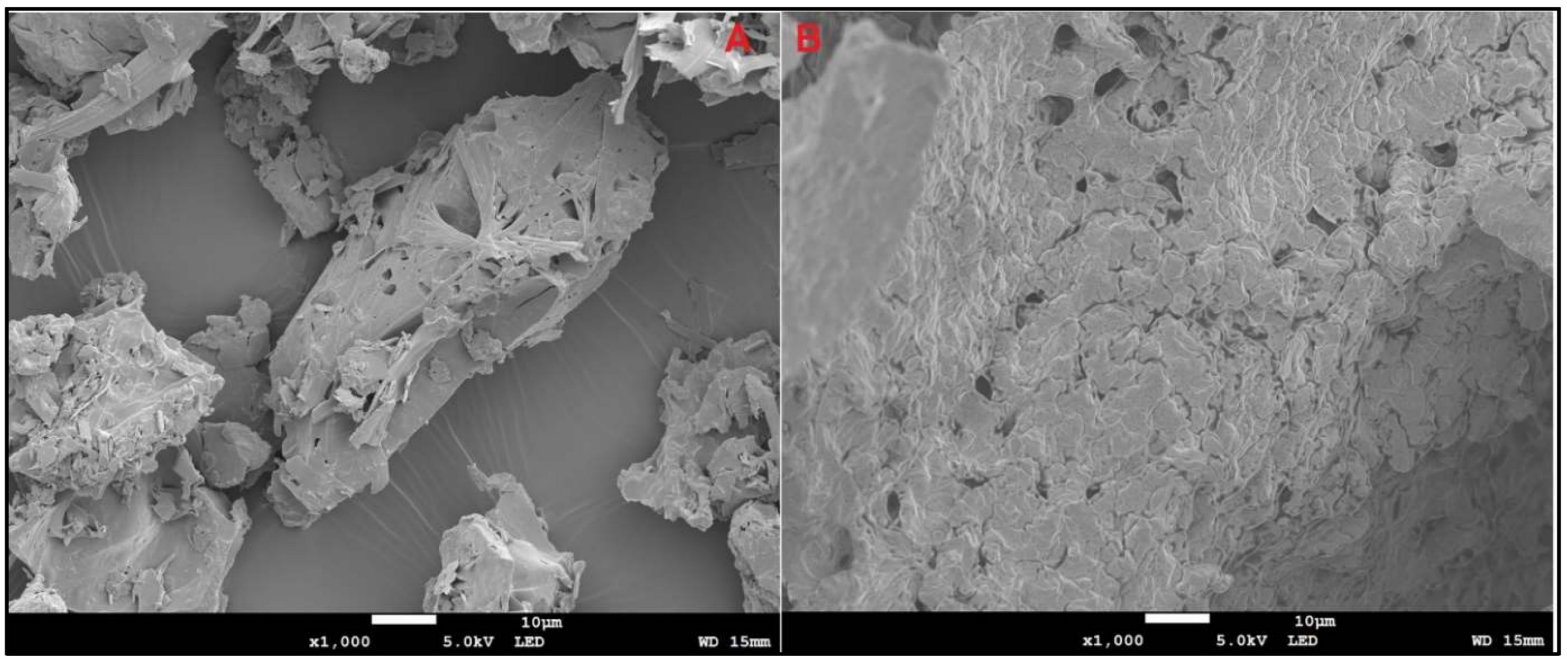
Scanning electron micrographs of Irbesartan (A) and IR-HPβCD inclusion complex (B).

The SEM micrographs in **Fig. 2 A and B** represent pure IR and IRHPβCD inclusion complex, respectively. A clear difference between the morphology of the IR and IR-HPβCD inclusion complex crystals is evident. The pure drug exists as flat crystals with a heterogenous surface, and irregular shape, whereas the IR-HPβCD inclusion complex shows a homogenous surface, indicating the inclusion complex formation. In addition, the IR-HPβCD inclusion complex demonstrated poor crystal structure, lacking distinctive crystal faces, and numerous fissures. However, the scanning electron micrograph of the physical mixture demonstrated that IR remained dispersed and physically adsorbed on the surface of HPβCD (image not shown), signifying no physical complexation. This could have contributed to the faster dissolution profiles, reported in our earlier investigation 18. The results were consistent with earlier published scanning electron micrographs.

### TEM

**Figure 3:**
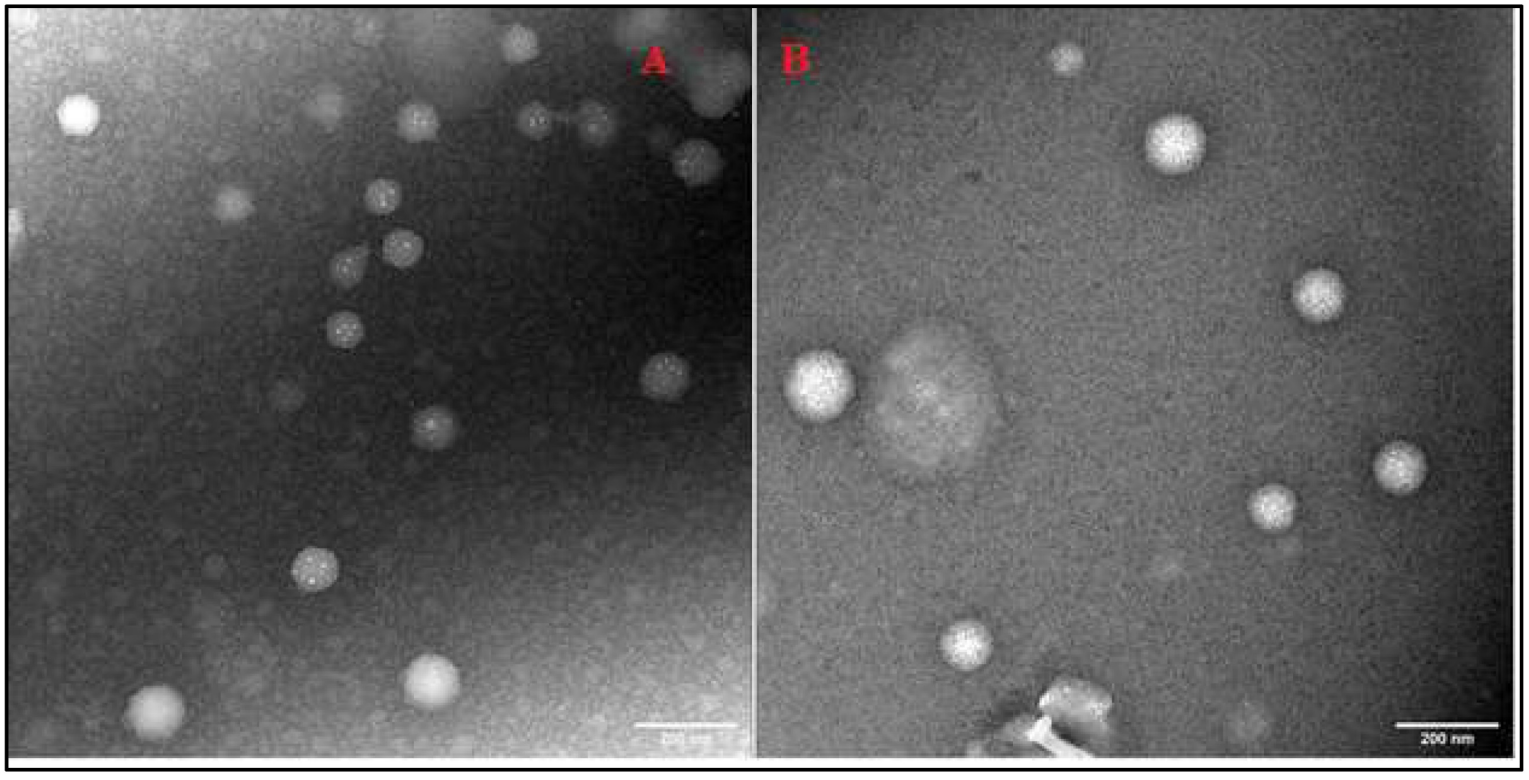
Transmission electron micrographs of IR-SLN (A) and IR-HPβCD-SLNs (B).

TEM can examine nanoparticles at scales as small as a single atom; therefore, TEM was used to analyze the morphology of SLNs. TEM analysis confirmed the spherical shape and colloidal sizes of the SLNs, detected by PCS (**Fig. 3 A-B**).

### *In vivo* PK studies

**Figure 4:**
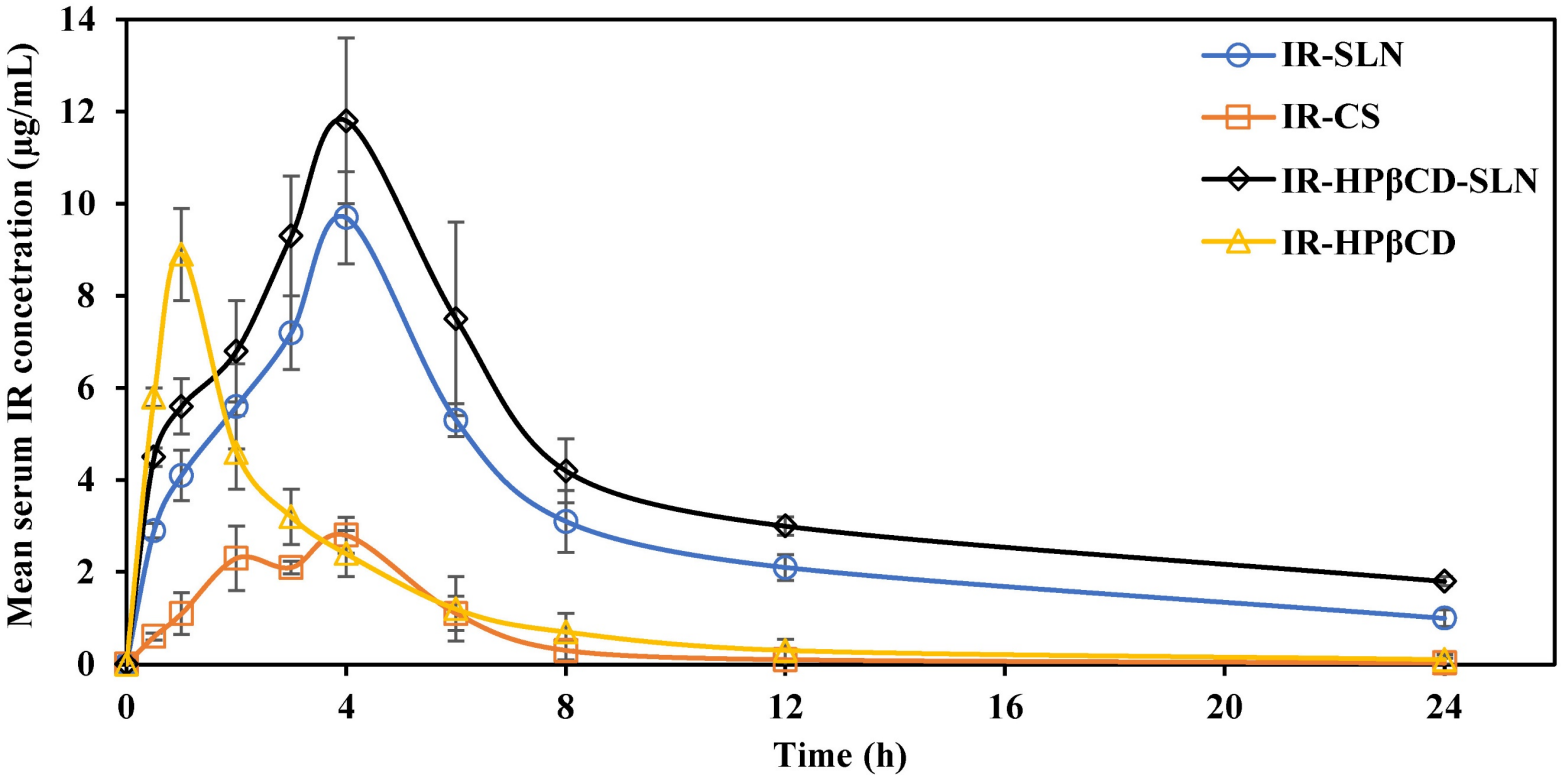
Mean serum concentration versus time plots of irbesartan following single oral administration of the SLN formulations (IR-SLNs, IR-HPβCD-SLNs), inclusion complex (IR-HPβCD), and suspension (IR-CS) in albino male Wistar rats (mean±SD; n=6).

IR mean serum concentration versus time plots after single oral administration of IR-SLN, IR-HPβCD-SLN, IR-HPβCD, and IR-CS formulations in albino male Wistar rats are depicted in **Fig. 4**. The relevant pharmacokinetic parameters (AUC_0→∞_, T_max_, C_max_, MRT, and t_1/2_) were calculated and are shown in **Table 3**. At all-time points during the study, the IR serum concentration was significantly higher for rats treated with IR-SLN and IR-HPβCD-SLN formulations than for those treated with IR-CS. However, the IR serum concentration of the inclusion complex was significantly higher than the IR serum concentration obtained from IR suspension during the first four hours only. Although there was no significant difference observed between the C_max_ (extent of IR absorption) of SLNs and inclusion complex formulations, the C_max_ values for the inclusion complex, IR-SLN, and IR-HPβCD-SLN were significantly higher (8.9±0.6 µg/ml, 9.7±1.0 µg/ml, and 11.8±2.1 µg/ml, respectively) than that observed for IR suspension (2.8±0.7 µg/ml). In addition, the AUC_0→∞_ value for IR-HPβCD-SLNs showed 1.5-, 4.7-, and 9.9-fold improvement in the relative oral BA of the drug compared to IR-SLN, IR-HPβCD, and IR-CS formulations, respectively. However, IR-SLNs showed 3.1- and 5.6-fold improvement in the relative oral BA of the drug when compared to IR-HPβCD and IR-CS formulations, respectively. Similarly, t_½_ of the SLNs and the inclusion complex formulations was longer than that obtained with IR suspension. Moreover, SLN formulations showed longer MRT than IR suspension, while the calculated MRT value of the inclusion complex was almost like the MRT value of the IR suspension. The slower elimination rate of IR from SLN formulations resulted in higher t_1/2_ and MRT values, and this observation confirmed the sustained release profile of IR from SLN formulations compared with the IR suspension and the inclusion complex.

IR has low oral BA due to low aqueous solubility, and it is also subjected to first-pass metabolism. Several mechanisms could have contributed to the increased oral BA of IR from SLNs such as: (i) the enormous surface area due to the nanosize of the SLNs could increase the rate of drug absorption, (ii) increased GI permeability by surfactants, and (iii) SLNs could enter the intervillar spaces due to small PS and prolong the residence time inside the GI tract and improve oral BA 25. Moreover, avoiding pre-systemic metabolism through uptake by the lymphatic system which is the major advantage of SLNs, is due to (i) small PS of nanoformulations (20-500 nm), (ii) surfactants enhance the uptake of nano lipid particles by Peyer's patches, and (iii) SLNs simulate the chylomicron structure with a more hydrophilic surface and a hydrophobic core 25,31,37.

Oral absorption for BCS class II drugs could be dissolution rate or solubility-limited, based on the solubility, dissolution, and permeability of the drug 38. If the *in vivo* dissolution rate of the drug is slower than the permeation rate through the GIT membrane, the oral absorption of the drug could be a dissolution rate-limited process 38. CDs can enhance drug delivery through aqueous diffusion-controlled barriers but not if the permeation of the drug through the biologic lipophilic barrier is the rate-limiting step 39. Therefore, the enhanced oral BA from the inclusion complex could be due to the rapid dissolution of IR. Based on these results, IR oral BA was improved significantly from all three prepared formulations compared with IR suspension.

### PD studies

Based on previously published investigations, oral fructose solution (10% w/v) was used to induce HTN in experimental animal models. Numerous earlier published animal studies have not reported a significant correlation between the short-term fructose-feeding (two weeks) and weight gain, demonstrating that the hypertensive effect is not linked to obesity in the used animal models 40,41. These studies have also revealed that high fructose diets can stimulate up-regulation of sodium and chloride transporters, sodium and chloride absorption, and consequently develop a state of salt overload that induce HTN 42,43. Moreover, excess fructose has been discovered to activate different vasoconstrictors, suppress vasodilators, and could over-stimulate the sympathetic nervous system 41. This observation was fortified further by our weight measurement results, which showed an increase in the individual rat weight by less than 6.1±0.9 % of the initial weight.

**Figure 5:**
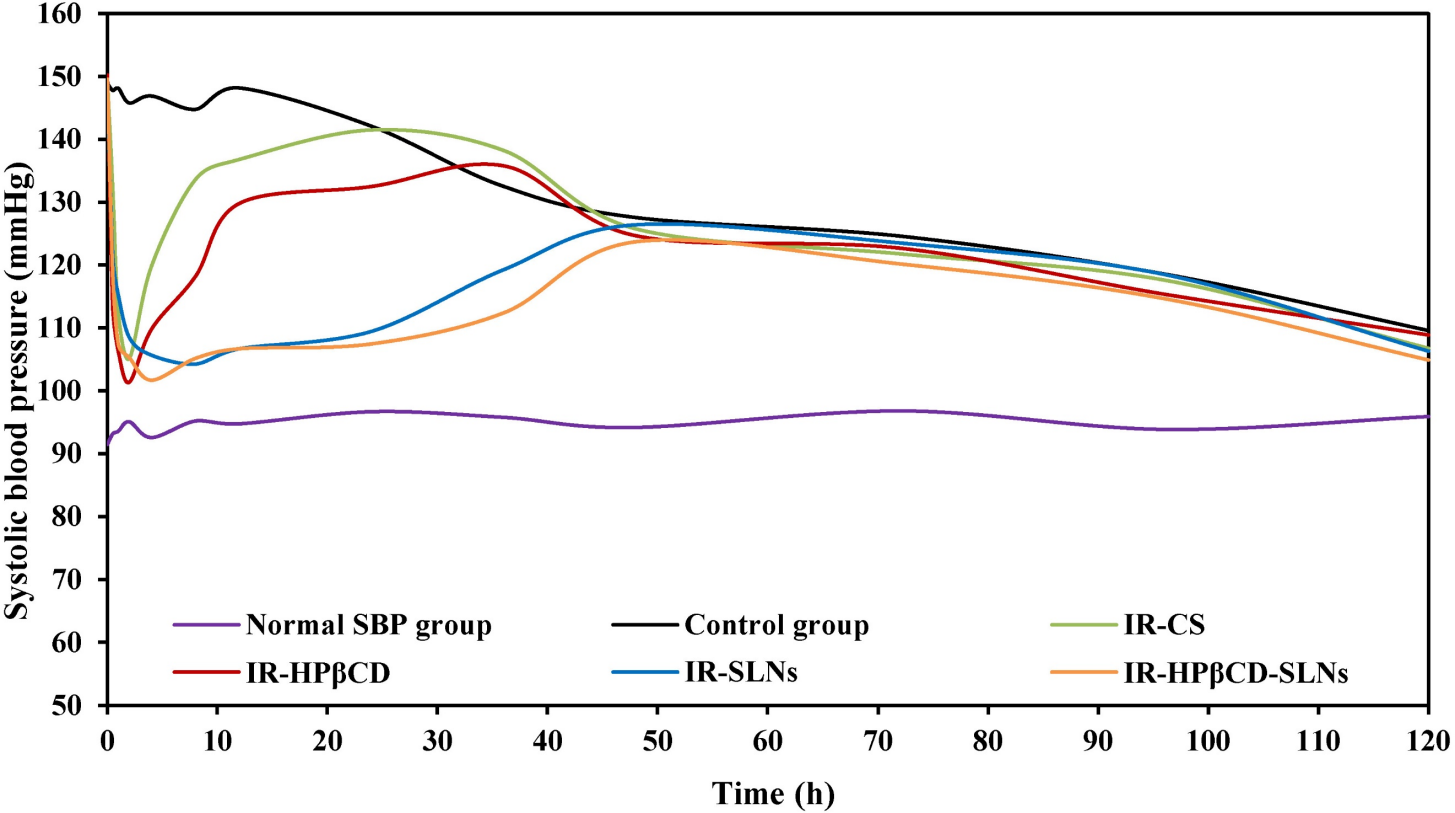
The antihypertensive effect of IR-SLNs, IR-HPβCD-SLNs, IR-HPβCD inclusion complex, and IR suspension (IR-CS) after oral administration in albino male Wistar rats (mean±SD, n=6).

The antihypertensive effect of the SLN formulations and the inclusion complex was studied in comparison to IR suspension in the albino male Wistar rat model by a noninvasive method. The SBP was measured based on the Tail-cuff technique and the results were depicted in **Fig. 5**. Group VI (untreated group) showed normal SBP during the PD experiment. IR-SLN and IR-HPβCD-SLN formulations resulted in an initial rapid decrease (p<0.05) in SBP during the first hour after oral administration, followed by a gradual decrease in SBP, with the maximum effect observed at 8 and 4 h for IR-SLN and IR-HPβCD-SLN formulations, respectively, and the effect of both SLN formulations continued for 48 h. However, the IR-HPβCD complex resulted in an initial rapid decrease (p<0.05) in SBP during the first 0.5 h of treatment, followed by a gradual decrease in SBP, with the maximum effect achieved at 2 h, but after 2 h, a gradual increase in BP was observed. By contrast, IR-CS significantly (p<0.05) reduced the BP initially, with the maximum effect observed at 2 h, but after 2 h, the BP started rising gradually until it was the same as the initially recorded value after 24 h. The control group showed no significant decrease (p>0.05) in the SBP up to 24 h after the induction of high BP due to the fructose solution. The drastic rapid decrease in BP within a short period (0.5 h) after oral administration of the inclusion complex could be due to rapid onset, revealed by short T_max_ (1.0±0.0 h) compared to IR-CS, IR-SLN, and IR-HPβCD-SLN formulations. Based on PK studies observations, the significant increase in t_1/2_ and MRT of SLNs in comparison to IR suspension and inclusion complex undoubtedly revealed that SLNs had sustained IR released and provided good control over SBP throughout 48 h and beyond. Thus, IR-HPβCD -SLNs revealed an effective approach to enhance the oral BA of insoluble IR and demonstrated a great potential for BP control over a longer period.

A couple of investigations have evaluated IR-loaded nanoformulations. The preparation and characterization (*in vitro* and *in vivo*) of IR-SLNs for HTN treatment, have been reported by *Soma et al*. 19. It was found that SLNs provided controlled release of IR for 48 h and exhibited good oral BA in Wistar rats in comparison to the IR suspension. However, the authors did not investigate/report PD studies for the nanoformulations in the experimental animals, and the EE values were low (41.2-73.8%). In another evaluation by *Shah et al*., researchers demonstrated a sustained release behavior of the prepared IR-SLNs; however, the *in vivo* performance of the formulation was not evaluated 44. The present investigation showed a comparative PK and PD evaluation of IR from the inclusion complex, SLNs, CD-SLNs, and IR suspension in experimental animals. To the best of our knowledge, this is the first study evaluating the effectiveness of CD-SLNs in the oral delivery of IR.

## Conclusion

Irbesartan delivery with a dual approach of CD complexation and loading into SLNs was successfully evaluated for developing an oral liquid formulation for irbesartan. The objectives of the current research, i.e., enhancement of solubility, avoiding pre-systemic hepatic metabolism, sustained delivery, and prolonged antihypertensive activity of IR were achieved. The observed results in the present investigation demonstrate the role of this approach as a promising vehicle for overcoming the shortcomings during the oral administration of BCS class II drugs. Therefore, the novel delivery approach revealed an effective therapeutic potential to provide good control over systolic blood pressure for up to 48 h and could meet the therapeutic needs of hypertension patients.

## Figures and Tables

**Table 1:** Composition of IR-SLN and IR-HPβCD-SLN nanodispersions.

Formulation	IR (mg)	IR-HPβCD (mg)	Dyanasn 112 (mg)	Soylecithin	Poloxamer 188 (mg)	Double distilled water up to (ml)
IR-SLN	10	---	200	200	150	10
IR-HPβCD-SLN	---	52.6^*^	200	200	150	10

*52.6 mg of the inclusion complex is equivalent to 10 mg of IR (Irbesartan).

**Table 2:** Physicochemical characteristics of IR-SLN and IR-HPβCD-SLN dispersions (mean ± SD, n=3).

Formulation	Size (nm)	PDI	ZP (mV)	Assay (%)	EE (%)
IR-SLN	244.3±5.9	0.22±0.05	-30.5±4.1	98.3±2.1	90.2±1.9
IR-HPβCD-SLN	261.5±3.9	0.23±0.05	-29.6±4.4	99.8±2.5	92.4±1.6

**Table 3:** Different pharmacokinetic parameters of irbesartan after single oral dose administration of SLN formulations (IR-SLN and IR-HPβCD-SLN), IR-HPβCD complex, and suspension (IR-CS) in albino male Wistar rats (mean±SD, n=6).

Parameter	Formulations			
	IR-CS	IR-HPβCD	IR-SLNs	IR-HPβCD-SLNs
C_max_ (µg/ml)	2.8 ± 0.7	8.9±0.6^*^	9.7±1.0^*^	11.8±2.1^*,$^
T_max_ (h)	4.0 ± 0.0^#^	1.0±0.0	4.0±0.0^#^	4.0±0.0^#^
AUC _total_ ((µg*h)/ml)	13.3±3.1	28.2±2.8^*^	87.3±4.7^*,#^	131.6±6.3^*,#,$^
T_1/2_ (h)	3.7±0.8	6.0±0.7^*^	10.0±1.3^*,#^	11.4±2.1^*,#,$^
MRT (h)	4.9±0.4	5.0±1.0	13.1±0.9^*,#^	16.0±1.3^*,#^

^*^Statistically significant at p < 0.05 in comparison to IR-CS. ^#^Statistically significant at p < 0.05 compared with IR-HPβCD. ^$^Statistically significant at p < 0.05 compared with IR-SLN.

## Abbreviations

IR: Irbesartan
HPβCD: Hydroxypropyl beta cyclodextrin
SLNs: Solid lipid nanoparticles
PK: Pharmacokinetics
PD: Pharmacodynamics
IR-HPβCD: Irbesartan complexed with hydroxypropyl beta cyclodextrin
IR-SLN: Irbesartan loaded solid lipid nanoparticles
IR-HPβCD-SLN: hydroxypropyl beta cyclodextrin complexed lipid nanoparticles of Irbesartan
HTN: Hypertension
USP-NF: The United States Pharmacopeia-National Formulary
EP: European Pharmacopoeia
BA: Bioavailability
βCD: Beta cyclodextrin
nm: nanometer
IR-CS: irbesartan suspension
rpm: round per minute
HPLC: High Performance Liquid Chromatography
M.W. cut off: Molecular weight cut-off
EE: entrapment efficiency
SEM: Scanning electron microscopy
TEM: Transmission electron microscopy
SBP: Systolic Blood Pressure
PDI: polydispersity index
ZP: Zeta potential
mV: millivolt
